# Exploring unfinished nursing care among nursing students: a discussion paper

**DOI:** 10.1186/s12912-023-01445-z

**Published:** 2023-08-18

**Authors:** Alvisa Palese, Stefania Chiappinotto, Aysun Bayram, Walter Sermeus, Riitta Suhonen, Evridiki Papastavrou

**Affiliations:** 1 Department of Medical Science, University of Udine, Udine, Italy; 2 Faculty of Health Sciences, Karadeniz Technical University, Trabzon, Turkey; 3 Leuven Institute for Healthcare Policy, University of Leuven, Leuven, Belgium; 4 Department of Nursing Science, University of Turku and Turku University Hospital, Turku, Finland; 5 Department of Nursing, Cyprus University of Technology, Limassol, Cyprus

**Keywords:** Unfinished nursing care, Missed nursing care, Rationed nursing care, Students, Discussion paper

## Abstract

**Background:**

In line with the impetus traceable among the nursing staff, studies regarding the perception of Unfinished Care among students have increased in recent years as also recommended by some policy documents in the consideration that, as future members of the staff, they are expected to raise concerns about failures in the standards of care. However, no discussion of their methodological requirements has been provided to date. The aim of this study is to debate Unfinished Care explorations among nursing students and developing recommendations.

**Methods:**

A Rapid Review was performed according to the Preferred Reporting Items for Systematic Reviews and Meta-Analyses, followed by a scientific discussion based on empirical evidence that emerged from the review combined with expert knowledge. Medline, the Cumulative Index to Nursing and Allied Health Literature (CINAHL), and Scopus databases were searched up to May 2022.

**Results:**

In the last five years, seven studies have been conducted by researchers affiliated at the university level, involving from 18 to 737 undergraduate students across Europe. By critically analysing their key aspects, there are derived some recommendations in conducting investigations in this field as, (a) the hidden meaning of Unfinished Care investigations among students by also deciding which concept is mostly appropriate to investigate; (b) the need of establishing alliances with the clinical settings in order to involve them in such explorations; (c) more complex research methods capable of exploring this issue among students by promoting learning outcomes and not only a simple data collection; and (e) the influences of these explorations on students’ wellbeing, as well as on ethical implications and that regarding the relationship between the healthcare services and the universities.

**Conclusion:**

Policymakers consider students to be key informants of the quality of nursing care issues witnessed during their clinical placements. The related emerging line of research is intriguing because of the underlying methodological, ethical and system complexities that need to be addressed according to some considerations.

**Supplementary Information:**

The online version contains supplementary material available at 10.1186/s12912-023-01445-z.

## Background

In line with the impetus traceable among nurses, studies regarding the perception of Unfinished Nursing Care among students have appeared in recent years [e.g., 1]. Unfinished Nursing Care has been defined as an umbrella or overarching term [2], including all of that established over the years as Task Left Undone, Missed Care, and Implicit Rationing of Care, and expressing any aspect of required patient care that is omitted either in part or delayed [3]. This emerging research is found in some policy documents [e.g., 4, 5] in the consideration that, as a future essential component of the workforce, students are expected to raise concerns about failures in the standards of care where patients are exposed to situations that could cause harm. The primacy of ensuring patient safety and of reporting concerns when the care is not in line with what is expected has been explicitly assigned to nurses by several professional codes across the world [e.g., 6, 7]. Some of them have included student nurses working with registered nurses in those cases where patient safety is at risk (Supplementary Table 1).

The role of students in detecting issues during their clinical education has been investigated from two main research angles over the years. The discrepancies between the expected care according to the theory learnt and that delivered in daily practice have been referred to as “reality shock” [8] or the traumatic impact felt by students when they experience this gap also according to its potential effect on patient’s safety [9]. In this context, the quality of the clinical environment, including the physical space, its pedagogical atmosphere, the ward manager’s leadership style, and the role of the nurse teacher, have been measured with different tools [10, 11]. This first research line is substantially student-centred and aims to investigate factors affecting the learning process [12]. Then, a second research line has been established involving students in assessing episodes of poor care as instances of neglect, incompetence, or abuse [13]. Similarly, suboptimal care [14] such as delays in treatments/diagnosis, poor assessment and pain management, unethical practices [15], and professional misconduct that violate patients’ rights [16] have also been explored among students. This second line of research can be also considered student-centred, as it aims to discover when and how students report poor care practices [17], their willingness to report issues [18] in the form of speaking-up or the reticence to report, as documented in the whistleblowing literature [16]. Alongside these lines, an additional approach is emerging connected with some policy-recommendations valuing the role of students in reporting lacks or issues as witnessed and/or experienced while experiencing the clinical rotations.

However, to our best knowledge, no discussion has been traced to date regarding which kinds of considerations are required when involving students in explorations related to the Unfinished Nursing Care. Students have been considered as a vulnerable population [19]; moreover, they attend clinical placements in health care settings, whereas their theoretical education takes place in the academic settings. Given this, the implications of such evaluations are complex as the quality of integration and collaboration between the academia and the health care services is not always clear [20]. In this light, the intent of this discussion paper is to debate Unfinished Care explorations among nursing students and to develop recommendations to improve the research in this field, as well as the related educational and management practices.

## Data sources

The study was designed in two steps: (1) a Rapid Review in eight phases [21] and (2) a scientific discussion based on empirical evidence that emerged from the Rapid Review combined with expert knowledge, as summarised in Table 1.

**Table 1:** Study steps: (1) Rapid Review and (2) discussion of empirical evidence with experts in the field

Step	Methods
**1. Rapid Review**	
**1.1 Needs assessment/analysis, topic selection, and topic refinement**	A first preliminary literature search aimed at informing the following steps and familiarizing with the topic, was performed. Some studies regarding poor care as perceived by nursing students have emerged (e.g., [17, 18]) with an impetus in recent years regarding the Unfinished Nursing Care perceptions among nursing students. Moreover, several policy documents have solicited the involvement of students in detecting poor care, episodes of neglected care, or similar issues (e.g., [4]). Therefore, to narrow the scope, the research team decided to perform a Rapid Review to answer the following questions: What studies have been conducted to date in the field of Unfinished Nursing Care as perceived by nursing students? What are their key methodological aspects?
**1.2 Protocol development**	The study protocol (not registered in a database) was designed by the researchers to address two main steps: (1) first, a Rapid Review was performed by adopting the Preferred Reporting Items for Systematic Reviews and Meta-Analyses [22] for literature search and findings report; (2) second, from the empirical evidence retrieved, researchers were engaged in a scientific discussion.
**1.3 Literature search**	The Preferred Reporting Items for Systematic Reviews and Meta-Analyses guidelines [22] were used. Three electronic databases were approached, namely Medline (through PubMed), the Cumulative Index to Nursing and Allied Health Literature (CINAHL), and Scopus up to May 2022. The following keywords were applied: “nursing students,” “missed nursing care,” “unfinished nursing care,” “rationing of nursing care,” and “prioritisation process.” “OR” and “AND” were used to combine all the keywords in each electronic database, and the search strings were changed as documented in Supplementary Table 2.
**1.4 Screening and study selection**	First, two researchers (AB, SC) performed the literature search. Then, one researcher (AP) worked independently to evaluate study eligibility based on keywords, title, and abstract screening of each study. In the second step, all eligible studies were retrieved in full text format, and then two researchers (AB, SC) independently read the full text of all articles and evaluated their inclusion. Furthermore, two researchers (SC, AB) also examined the grey literature (no items were found) and references of included studies were screened manually. Any differences regarding eligibility were discussed with the remaining members of the team (see authors). All primary studies that were written in English involving nursing students at all levels of education that investigated Unfinished Nursing Care in all its possible terms in any study type (qualitative, quantative, thesis, etc.), except for systematic reviews, reviews, and books, were included. The processes of study selection and inclusion are reported in Fig. 1 [22].
**1.5 Data extraction**	A data extraction grid that was developed and piloted with two of the included studies. Then, two researchers (AB, SC) extracted the following data from each included study: (1) author; year of publication; country; affiliation (e.g., university); (2) aims; study design; setting; year of data collection; (3) sample and participants, including response rates, and participants’ main characteristics; (4) data collection process; and (5) main findings. The researchers worked independently and then compared the extracted data. Differences, if any, were discussed with a third researcher (AP) until full agreement was reached.
**1.6 Risk-of-bias prevention**	To prevent bias, some strategies were applied: (1) a preliminarily literature search was conducted by two researcher (SC, AB); (2) three researchers were involved in the definition of the inclusion and exclusion criteria (SC, AB, AP); (3) three researchers were involved in the literature search, study selection process, and data extraction; (4) structured guidelines were used in the study process and reporting; (5) data extraction was completed with verification by all researchers; and (6) a consensus was sought among the researchers to move on to the next process/stage.
**1.7 Knowledge synthesis**	A narrative summary of the methodological aspects and findings of the retrieved studies was performed. These were summarised (a) the country where the study was conducted; (b) the affiliation of the author(s) (e.g., University, Hospital); (c) the main aims of the study; (d) the underlying concept or the conceptual framework considered (e.g., missed nursing care, implicit rationing of nursing care); (e) the study design; (f) the sampling methods by also summarising the inclusion and exclusion criteria as well as the participants profile, and participation rates; (g) the data collection procedures and the tools used; (h) the main aspects investigated (e.g., the meaning given by students to the phenomenon, themes and sub-themes); (i) the ethical approval and considerations reported in the study, and (l) the main findings.
**1.8 Report production and dissemination**	The findings were documented and a draft prepared with the key issues. Then, the draft document was sent to all researchers, and they were invited to read it, as an individual reflection; after two weeks, the suggestions were shared among the team and the step 2 began.
**2. Discussion of empirical evidence with experts in the field**	
**2.1 Discussion process**	With the intent to summarise the key considerations required while involving students in Unfinished Care explorations, researchers were involved in multiple rounds where inductive (from the evidence emerged) and deductive (by developing specific predictions from general principles, [43] reasoning, was conducted: each limitation, potentiality, and recommendation were first labelled, then described in its contents and provided with an example. The process was conducted by starting with a draft and collecting feedbacks and incorporating them progressively. Disagreements were also discussed, and the refined document was approved by all researchers (see authors).

Briefly, according to the findings of a preliminarily need analysis, two research questions were established: (a) What studies have been conducted to date in the field of Unfinished Care as perceived by nursing students? (b) What are the key methodological aspects and/or implications of these studies?

Then, as described in detail in Table 1, the Rapid Review was designed and developed according to the Preferred Reporting Items for Systematic Reviews and Meta-Analyses [22]. Medline (through PubMed), the Cumulative Index to Nursing and Allied Health Literature (CINAHL), and Scopus databases were searched up to May 2022 by two researchers (SC, AB) with the following keywords: “nursing students,” “missed nursing care,” “unfinished nursing care,” “rationing of nursing care,” and “prioritisation process” (Supplementary Tables 2 and 3). All primary studies (e.g., qualitative, quantitative) written in English, involving nursing students at all levels of education, and investigating Unfinished Care in all possible terms, were eligible. The reference list of retrieved studies was also screened by two researchers (SC, AB).

Then, all included studies were carefully read, and data relevant to the research questions were extracted using a grid developed and piloted by researchers (AB, SC) in two studies. The main features of the studies retrieved were then summarised and analysed individually and then together, by the research team (Table 1). The aim was to provide recommendations in this research field, as well as in the educational and managerial practices by combining empirical evidence and expert opinions. Specifically, the researchers were involved in inductive (from the evidence retrieved) and deductive reasoning (the process of developing specific predictions from general principles [23]), where recommendations were first labelled as a proposition, and then fully described.

## Results

**Fig. 1:**
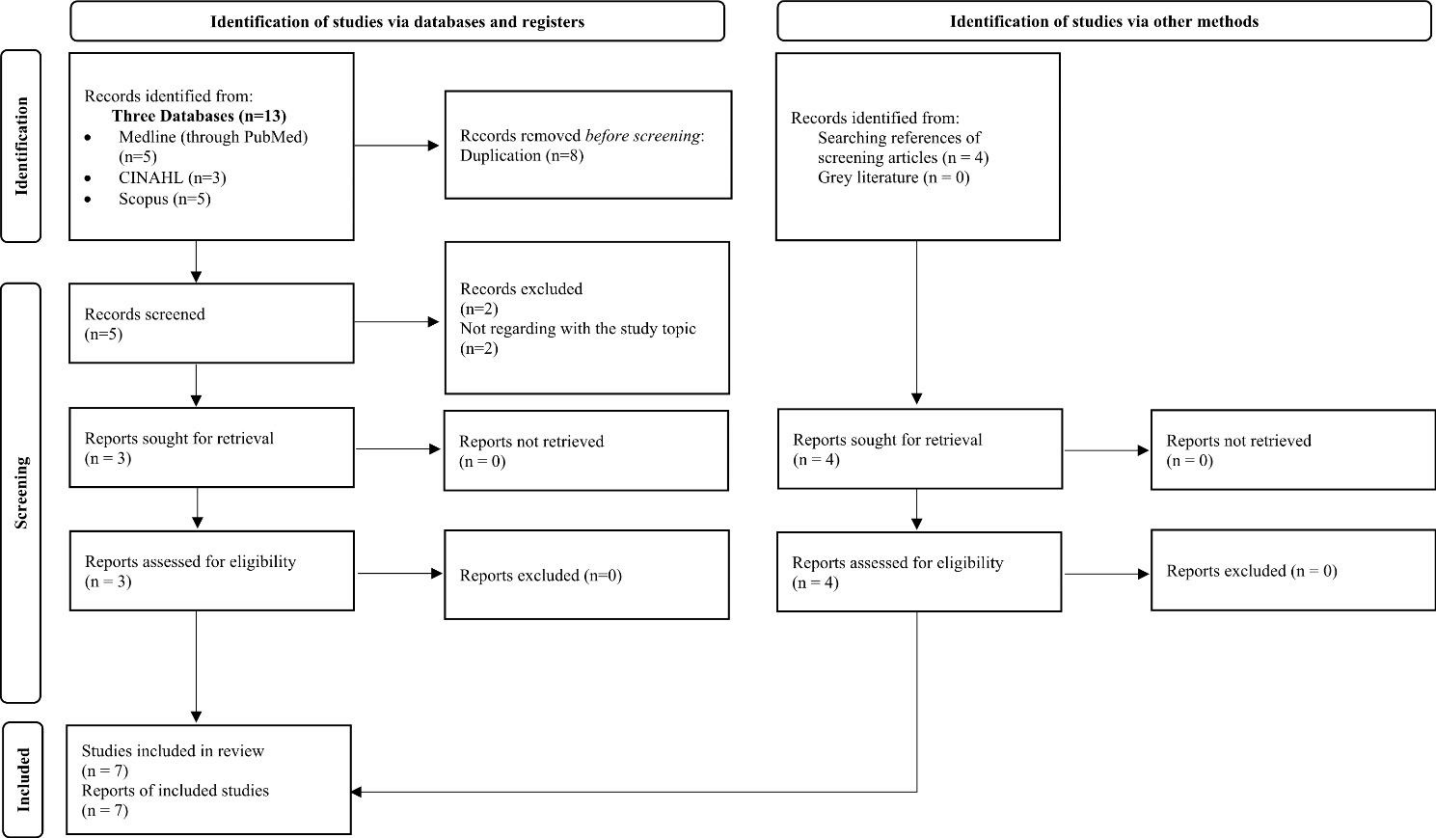
Flow diagram for systematic reviews which included searches of databases, registers and other sources **LEGEND**: CINAHL: Cumulative Index to Nursing and Allied Health Literature

The seven studies retrieved (Fig. 1) were published from 2017 to 2022 by authors with university affiliations (Supplementary Table 4). As summarised in Table 2, among the six qualitative studies, Kalfoss [1] investigated the meaning and causes of missed care as perceived by 32 postgraduate students working in different fields in Norway by performing an explorative qualitative design with focus groups. Several constraints were perceived as triggering missed care at the labour, organisational, professional, and communication levels, impacting on the students emotionally, threatening their sense of power and identity, and leading to irritability/fatigue. In the same years, Gibbons and Crane [24] performed a qualitative study in the United Kingdom to explore how missed care influences students’ socialisation. In this study, 18 undergraduate students were invited into two focus groups that used a scenario format. All participants were aware of missed care and have been socialised to pragmatically accepted it as the norm.

**Table 2:** Key aspects of the studies included in the Rapid Review

Key aspects	Kalfoss, 2017 [1]	Gibbon & Crane, 2018 [24]	Najafi et al., 2021 [25]	Kalánková et al., 2021 [26]	Habermann et al., 2022 [27]	Dimitriadou et al., 2021 [9]	Palese et al., 2021 [28]
**Country**	Norway	UK	Iran	Slovakia	Germany	Greece and Cyprus	Italy
**Affiliation of authors**	University	University	University	University	University	University	University
**Main aims**	Perceptions of missed care and contributing factors	How exposure to missed care influence students’ professional socialisation	Lived experience of missed care	How nursing students interpret rationed care and their experience	Lived experience of missed care	Perceptions of missed care occurrence, reasons, outcomes	Tool validation
**Areas explored**							
*Phenomenon meaning*	√		√		√		
*Units mostly affected*		√		√	√	√	√
*Perceived causes*	√				√	√	√
*Students’ decision-making process*		√			√		
*Students dealt with it*					√		
*Perceived implications*		√	√	√	√	√	
**Underlying concept**	Missed Nursing Care	Missed Nursing Care	Missed Nursing Care	Rationed Nursing Care	Missed Nursing Care	Missed Nursing Care	Unfinished Care
**Study design**	Explorative qualitative	Qualitative	Interpretative phenomenology	Qualitative	Qualitative	Inductive content analysis	Validation study
**Participants** **Students’ level**	Postgraduate	Undergraduate, final year	Master’s degree students	Undergraduate, final year	Undergraduates, from 1st to 3rd year	Undergraduate, 3rd to the 4rd year	Undergraduates, from 1st to 3rd year
**Sampling**	Purposeful	Participants invited and those interested involved	Purposeful	Purposeful	Purposeful	All students of five universities	All students of three universities
**Participants and response rate**	32/32 (100%)	10 + 8 (NR)	10/10 (100%)	18/18 (100%)	69/69 (100%)	229 (Cyprus) (NR) + 381 (Greece) (NR)	737 (61.9%)
**Data collection procedure**	Six focus groups	Two focus groups	Individual, in-depth, face-to-face interview	Individual, face-to-face interview	Written online reports	Open-ended questions in a survey	Tool with closed-ended questions
*Examples of questions/items*	What do you think about…? Can you give some examples…?	The problem you just read in the scenario are very similar to that reported by nurses and literature…	What comes to your mind when I say missed care? How do you feel?	Student experience with elements of care regularly rationed Reasons	Phenomenon definition, examples experienced, how they dealt with it	What are missed care events witnessed in the practice? What are the reasons and the impact?	e.g., Mouth care; how often omitted/delayed (or witnessed nurses omitting/delaying) ‘always’ - ‘never’ Causes: e.g., tensions among nurses; ‘not a significant’- ‘significant reason’
**Ethical/IRB approval**	Yes	Yes	Yes	Yes	Yes	Yes	Yes

**LEGEND**: IRB: Internal Review Board; NR: Not reported; UK: United Kingdom

Najafi and colleagues [25] conducted an interpretative phenomenological study in Iran involving 10 master’s degree students in individual face-to-face interviews to explore their lived experience regarding missed care and to perform a reflective exercise to prepare future leaders to deal with this issue. Missed care was considered unfilled care, leading to serious consequences for patients, including death. Students experienced several negative emotions when engaged in or observing missed care episodes, including ethical conflicts that continued after the shift was over.

In the same year, in Slovakia, a qualitative study was performed by Kalánková and colleagues [26] with the intent of exploring how students interpret and experience rationed care during clinical placements. Eighteen full-time undergraduate students in their final year were interviewed individually. According to the findings, incomplete care was normalised. Priority was given to tasks ordered by doctors; care was ritualised and when workloads were high, care was impersonal. The pressure of these implicit norms and the fear of consequences prevented students providing the best care.

Habermann and colleagues [27] also performed a qualitative study in Germany involving 69 students drawn from the first to the third years who were asked to write a report on missed care they might have experienced during clinical placements. They reported a range of unfinished care, as omitted, partially omitted, or delayed; these episodes triggered negative feelings, impacting on their learning opportunities, professional standards, student status, and on patients. Different strategies were adopted by students to deal with missed care, from reporting to mentors, asking for help, accepting the situation, speaking with friends, applying self-management strategies, and speaking with patients/residents.

More recently, Dimitriadou and colleagues [9] explored third- and fourth- year undergraduate students’ perceptions regarding the reasons and consequences of missed care witnessed in their clinical placements. Open-ended questions collected in a survey involving 229 students in Cyprus and 381 in Greece were analysed. Nurses’ indifference, lack of interest and knowledge regarding the care required, and high workloads were reported as causes, with several negative impacts reported also on patients (e.g., nosocomial infections).

Only one quantitative study [28] has been published in this research field to validate a tool measuring the occurrence and causes of Unfinished Care among students. This study involved 737 Italian students in their first to final year attending clinical education in a hospital or community settings. The Unfinished Nursing Care Survey for Students was validated in terms of acceptability, construct validity, hypothesis testing, and criterion validity as composed of parts A (22 items, elements of care) and B (18 items, causes).

## Discussion

### Empirical evidence available

In the last five years, seven studies have been conducted by researchers affiliated at the university level, mainly relying on missed nursing care as a construct, involving from 18 to 737 students, and attending their undergraduate education across Europe. Of the seven included, only one resulted to be quantitative as a validation study [28], while the majority were qualitative, and this might be explained under different angles. Firstly, researchers attempted to gain and deepened the knowledge regarding the students’ experience, to merge the main issues; secondly, this research field is in its infancy and validated tools are unavailable; thirdly, Unfinished Nursing Care issues are sensitive topics thus better explorable with qualitative approaches.

Overall, the studies have deepened the (a) meaning of Unfinished Care, (b) its occurrence, (c) causes, (d) the decision-making processes enacted by students, (e) how they cope with it, and (f) the implications for patients and nurses, as well as the consequences for students’ socialisation, dilemmas, and feelings. A part their intrinsic value as investigations, these studies have contributed in understanding the factors perpetuating Unfinished Care [29] according to the shaped attitudes formed during students’ clinical rotations, where professional socialisation takes place. Moreover, engaging students early in this discourse, with qualitative approaches according to their “*good position to spot things that might be wrong*” [4], may have increased their responsibility for action, promote their positive self-perception, and avoid the passive perception that “*I am only a student*” [30]. Furthermore, involving students in detecting episodes of unfinished care may have prepared them to navigate the barriers affecting nursing care [31]. Their involvement might also function as an educational strategy [18] to empower them and to challenge the idea that missed care ‘is normal’ [28].

### Considerations required while involving students in unfinished care explorations

*Reflecting on the hidden meaning of Unfinished Care investigations among students*. Measuring the frequency and nature of Unfinished Care episodes encountered by students, and when and where they encounter them, is important in promoting quality [18]. Acknowledging that missed care endangers patient safety is an important step in improving their care. Witnessing and reporting/discussing poor care might also have an educational function [13] as a concrete example of what should not happen, and as a source of moral reflection in changing practice for the better. However, insisting on these explorations and measures might legitimate and emphasise that poor care exists, focusing the attention on the lack instead of the potentialities of nursing care. Educators should highlight examples of good practice and encourage students to remember that Unfinished Care reflects episodes that are not the norm [31].

*Declaring the underlying concept of the study*. Unfinished Care has been considered an overarching term [2], including all different terminologies used in this field (e.g., missed care, rationed care). However, despite the inclusion of all these terms under one term, the different concepts imply different interpretations of the processes and causes of this phenomenon, as for example, the relevance of the habits in missed care [3] versus the relevance of implicit rationing in rationed care [32]. These differences may be important for students to consider, as although they lead to the same outcomes as omitted or delayed care, the underlying causes or factors differs. Therefore, it is important that future studies continue to specify the underpinning concept to conduct theoretically sound investigations.

*Establishing alliances with the clinical settings*. Studies available have not explicitly reported the collaboration with the clinical settings; all were based at the university level, triggering three main considerations:

- First, reporting issues internally (to the nurses) or externally (to a nurse researcher/educator) may have different implications. Clinical nurses are more confident in reporting issues internally; for students, it may be easier to report externally, although available guides have recommended that before contacting any regulatory body or other external institution, they should follow protocols and take advice from their preceptors [e.g., 33]. Universities/Higher Education Institutions (HEIs) are encouraged to offer education regarding how “*raising concerns*” with protocols/algorithms in agreement with their practice partners as part of the accreditation process. Mentors and facilities need to be informed that nursing students are taught how to raise concerns about care [33]. However, students should be invited to raise concerns externally (also to researchers) when all other procedures have been followed.
- Second, emphasising only the etic perspective (= that coming from outside, researchers/students) may limit the evaluation to a mere research exercise. In contrast, ensuring the emic perspectives (= involving insiders, such as clinical nurses and nurse managers [34]) might promote a strong alliance between health care services and universities/HEIs that enhance patient and student safety. This might increase the likelihood of promoting intervention studies to improve the situation [e.g., 35], gain insights from different perspectives, ensure clinical nurses/nurse managers that data are collected under a bilateral agreement, and ensure that students’ involvement is valued by both parties. Cooperation between health care institutions and universities/HEIs has been underlined as critical [36]; in this context, measuring Unfinished Care unilaterally might increase the distance between the academy and the clinical settings.
- Third, students report issues when they perceive the support of the clinical environment [16]. Establishing alliances between institutions may create a sense of security and normality in reporting issues. However, the student–mentor relationship is one of the key factors influencing students’ willingness to report potentially unsafe practices [31]. When students are not exposed to role models who encourage the reporting of poor practice it may be of benefit to use external surveys. This should be considered the last option, given that clinical placements with negative role models should be avoided.

*Identify and address new challenging aims.* Available studies have mainly investigated the occurrence and consequences of Unfinished Care according to students’ “*fresh*” pair of eyes [4], as they are in the best position to detect care issues normalised in practice [31]. However, available data shows that they detect the same level of Unfinished Care [28] as their preceptors, suggesting that they readily lose their “*fresh pair of eyes”*. If this is confirmed by future studies, the investigation of Unfinished Care among students should be adjusted to the more important steps concerning:

- Factors involved in deciding priorities leading to Unfinished Care. Evidence suggests that students are socialised to prioritisation skills in the early stages of education [29]. Therefore, understanding how they shape these skills and how they can be effectively trained is crucial.
- How students develop a sense of understanding of the invisible part of nursing care. Students may report data about the practice they witness that is visible in its behaviours without understanding the underlying decision-making process [31]. Coaching students to openly ask their clinical mentors the underlying reasons for the decisions undertaken (e.g., to postpone an intervention) might increase their understanding of the nature of the deviance, helping them go behind what they observe. Moreover, coaching them to identify minor concerns can prevent more serious and perhaps life-threatening issues [33].
- Go beyond the available evidence. Unfinished Care is considered a matter of low staffing and resource levels, and students might be convinced that with an increased number of nurses, the issue will be resolved. Future investigations should consider that Unfinished Care can be considered also as a form of marginalisation, discrimination, and inequality in care and service delivery [26]; moreover, as a side effect, neglected needs, rights abuse or violations, wrongdoing/misconduct, and failures to commit good quality care cause nurses to leave the profession.
- Reasons leading to Unfinished Care and strategies to overcome it. Some studies have not investigated the causes of Unfinished Care because students are in a precarious position or are not experts. However, the same reasons documented among nurses [37] have been reported by students [28], suggesting that students may contribute to also understanding the causes from other perspective as that reported by the nurses. However, behind the causes, suggestions regarding strategies to overcome the phenomenon might be important to investigate to prepare students to deal with issues in their professional lives.

*Reflecting on the impact on the student’s wellbeing.* The impact of patient safety incidents (‘*primary victims’*) on the wellbeing of caregivers is well researched. As most of these incidents are not only related to individual mistakes but also to system failure, the caregivers involved in such an incident are often called “*second victims*.” Experiencing the second victim role, has been reported to lead to higher burn-out and turn-over [38]. It might be important to investigate what the impact is on students’ personal wellbeing if they are involved in systematic rationing of nursing care, and on its external reporting.

*Integrating qualitative and quantitative approaches.* Available studies have mainly used qualitative approaches based on reflections and shared experiences according to the lack of visibility of the phenomenon, the need to increase our understanding of it, and the capacity of these reflections to promote learning. However, quantitative measures may help in quantifying a phenomenon, and compare its frequency over time and across settings. While qualitative studies personally involve students to reflect on their own experience, individually or in group, quantitative measures position students as “*external evaluators*.” Furthermore, survey (e.g., via online) resulting in quantitative findings are not discussed in depth; thus, they may reflect a collection of sterile data far from a learning occasion. Therefore, interpretive studies or mixed-method studies employing both approaches are suggested to enhance the learning intent of the investigation and thus the benefit for students in deepening their understanding of nursing practice and the benefit to measure the phenomenon. In studies based only on quantitative surveys, creating occasions to reflect on findings with students and the nursing staff of the settings can transform a pure investigation into a source of learning and improvement.

*Deciding when and who should be involved*. There are different trends in studies available in line with their aims and designs, from involving across all years or using purposeful samples, from undergraduate and postgraduate students. These decisions should be weighed according to the following considerations:

- In the early stages of the programme, students may not be able to identify Unfinished Care episodes. First-year students report lower levels of Unfinished Care, while a higher occurrence has been documented by second-year students and decreases among third-year students [28]. First- and second-year students have little clinical experience, and they might be more attached to learned theories [39]. In contrast, third-year or final-year students have attended different clinical rotations; their exposure to the care is different both in quantitative and qualitative [e.g., different settings; 40] and they might see unfinished care as normalised. However, they have transitioned from one clinical placement to the next and are thus in the position of being able to compare different placements and detect care issues. Paradoxically, mature students, such as those attending postgraduate courses, have been less involved in studies. Given their future advanced roles, involving them will have an important impact [25].
- At the overall level, to provide credible evaluations, it is important to consider students’ competences and safety knowledge, and/or give them the option (in quantitative measures) of “*I don’t know*” or “*I do not have sufficient knowledge to evaluate this/I have no experience*.” There is a need to be sure that students understand that care omissions or delays are unacceptable; however, they may perceive or not omission, according to their level of education. Therefore, they should be motivated and educated to report their perceptions, that may change over time according to their ability to recognise Unfinished Care episodes.
- Available studies have mainly used purposeful samples, which means that students are considered key informants in the phenomenon of interest. The reasons why they are considered key informants (e.g., because of their ability to critically appraise the practice, their recent clinical placement in a critical setting, or their ability to report issues to nurse educators) should be documented in future studies. In contrast, a few studies involved all students, which has several implications. Not all students might be aware of Unfinished Care given their level of education; moreover, not all will participate: in fact, a participation rate of 61.9% [28] has been documented in line with that reported among nurses [28]. Students may be burdened by several questionnaires (e.g., the quality of lessons, that of clinical placements), but they may also be reluctant to respond due to the ethical dilemma [15] of reporting outside of the unit the issues, or a lack of confidence in disclosing failures in nursing care. In addition, not answering a survey, deciding therefore to be silent versus being a whistle-blower of patient care neglects, might also be due to the fear of being identified.

*Deciding instruments and methods of data collection*. Available studies have used three main strategies to collect data: the majority have asked concrete examples witnessed by students [e.g., 1], one study used a survey tool [28], and one provided a scenario [24]. Of course, each strategy has its methodological potentialities and limitations, but it should be considered for the implications in the context of nursing education. Reflecting on the witnessed care is typical in debriefings sections; however, in a research context, asking to report witnessed practices outside of the unit implies that these practices are already discussed inside of the unit with the clinical nurse or the nurse manager. Differently, collecting data with tools should consider the validity of the measures among students and which concept of nursing they reflect on. Tools used up to date need to be further developed, refined, repeatedly used, and validated to overcome their task-oriented approach [28]. In addition, triggering the discussion with a scenario-based measure allows neutrality, where students are not forced to report outside what they have witnessed allowing them to preserve ethical principles.

*Considering the implications of time*. In general, the available studies have not emphasised when episodes of Unfinished Care occur. In this regard, at least two main factors should be considered:

- Reporting in a tool, in a focus group, or in an individual interview, the unfinished care witnessed after a certain time may increase clarity and lucidity; however, when the time elapsed between the episode and its reporting is significant, recall bias might affect the quality of the information, and the perception to contribute to the practice change might be prevented, making reporting useless. In an ideal world, students should be coached to speak up with assertive communication regarding clinical situations requiring (immediate) action(s) to resolve an issue and should be encouraged to decide whether immediate action is required before participating in Unfinished Care studies.
- Students need time to capture the global picture of the quality of care in each context; therefore, studies should report when data collection occurred, the duration of the clinical placement and at what stage in the student’s education it occurred as these factors influence students’ critical evaluations.

*Reflecting on ethical implications.* All retrieved studies were approved by an ethical committee, and this is important for the ethical implications of this kind of research itself, given that students are called on to share issues of the practice outside of the setting. In all cases, anonymity was ensured, which may be a strategy to protect students as vulnerable participants. However, the question of whether learning by doing is still valid in the context of Unfinished Care is important, given that students aspire to the best care, which may cause some problems without critical thinking. Therefore, this kind of research may offer an occasion to learn that an ethical dimension is embodied in all activities [41], how to apply ethical values versus accepting unethical practices, and how to speak up when patient care is neglected [15].

However, according to the research process documented in the studies retrieved, these have not considered the commitment to return the findings (and to discuss them) to the students and/or the clinical settings; this also might have ethical implications and could be important in the future in encouraging students to continually promote the practice and to believe that their evaluations are important to promote its change.

*The need to assess certain preconditions.* Researchers, educators, and managers should ensure that studies or evaluations involving students in the assessment of Unfinished Care is based on certain preconditions. First, students need to be trained to speak openly about issues encountered in practice with their clinical nurses/nurse managers and to recognise the level of quality of care and its minimum standards. Second, the clinical environment should provide systems/processes to follow when observing or suspecting wrongdoing/sub-standard care and should include omissions/care left undone into patient safety risk incident reporting. Third, an alliance between health care settings and universities/HEIs is needed so that students perceive the usefulness and the collaborative intent of their involvement in studies and that their observations are not merely a research exercise.

In designing studies in this field, more emphasis on examples of good practice (and not only on issues) is recommended. There is also a need to challenge new ambitious objectives, avoiding the replication of those already achieved, in order to also understand the mechanisms by which students shape their decision-making prioritisation [42] perpetuating Unfinished Care. Furthermore, according to the complexity of this research, mixed-methods studies are encouraged, carefully considering which students to involve, how, and when, and weighing up the benefits and risks. Establishing that findings will be returned and transparently discussed with the clinical settings and their main actors, may transform this kind of research from merely descriptive into a concrete opportunity for improvement and learning across systems (health care service and University/HEIs) and their main actors.

### Limitations

We have conducted our investigation by combining two different study designs, a Rapid Review, and a discussion of empirical evidence with experts in the field. In the Rapid Review, we considered only studies investigating the issues among nursing students: according to the intent which was to stimulate the discussion and not to summarise the evidence available in the field, no quality assessment of retrieved studies was conducted, and a selective process of data extraction was applied. Moreover, only primary studies were included considering (a) the recent debate regarding the topic; (b) the intent to extract and debate the empirical evidence as reported in primary studies. In the second step, the discussion was developed involving internationally recognised experts (AP, EP, RS, WS) and younger researchers (SC, AB) in the field. The debate conducted on distance and not in person, as well as the non-anonymity of the feedback progressively incorporated, might have influenced the findings. Moreover, the discussion, was not guided by a structured approach and this might also have introduced some influences. At the overall level, the scientific discussion has been conducted mainly around the key methodological aspects that emerged from available studies (Table 2); in the future, in-depth reflection regarding some specific elements (e.g., ethical issues while involving students) should be promoted.

However, while considering the study limitations, there are no previous discussions and recommendations for research in this field in the literature, to significantly support future studies on Unfinished Care among nursing students.

## Conclusion

Recently, policymakers have considered students to be possible key informants regarding issues in the quality nursing care witnessed during their clinical placements. In line with well-established research on Unfinished Care among nurses, a new line of research has recently started its course with studies generally conducted in Europe, that are mostly qualitative and involve undergraduate students. The emerging line of research is intriguing for the underlying methodological complexities and needs to be developed around some considerations. Overall, there is a need to avoid using students only for research purposes and to transform their involvement into a learning opportunity, capable of (a) promoting quality improvements, (b) increasing their readiness as future nurses to prevent and act towards Unfinished Care, and (c) avoiding its normalisation in the practice. Alliances between Universities/HEIs should be promoted to value students’ evaluations in the field of Unfinished Care.

### Electronic supplementary material

Below is the link to the electronic supplementary material.

**Supplementary Table 1**. Nursing students and poor care reporting: an example of professional guidance extended to nursing students

## Data Availability

All data generated or analysed during this study are included in this published article [and its supplementary information files].
