# Supplementary material for: Exploring unfinished nursing care among nursing students: a discussion paper
Source: BMC Nurs. 2023 Aug 18;22:272. doi: 10.1186/s12912-023-01445-z (PMC10436392; doi:10.1186/s12912-023-01445-z)
Supplement: Supplementary file 1 — Supplementary Table 1. Nursing students and poor care reporting: an example of professional guidance extended to nursing students [file 12912_2023_1445_MOESM1_ESM.docx]

**Supplementary Table 1.** Nursing students and poor care reporting: an example of professional guidance extended to nursing students

In the UK, since April 2015, whistle-blower protection has been extended to students. The Raising Concerns Guidance for nurses, midwives, and nursing associates [1] supports and should be read together with The Code: Professional standards of practice and behaviour for nurses, midwives, and nursing associates [2]. Actions should be undertaken in situations of neglect and acts of omission.

The guidance is for all nurses, midwives, nursing associates, and pre-registration nursing and midwifery students. Principles should be applied in the same way, and in the case of students, specific indications are offered, for example:

- Inform your mentor, tutor, or lecturer immediately if you believe that you, a colleague, or anyone else may be putting someone at risk of harm.

- Seek help immediately from an appropriately qualified professional if someone you are providing care for has suffered harm for any reason.

The guide recommends that students “*talk to your university tutor or lecturer, your mentor or another registered nurse, midwife or nursing associate in your practice area*” [2]. Moreover, students are encouraged to follow employer’s policies on raising concerns or whistleblowing.

[1] Nursing & Midwifery Council. *Raising concerns: Guidance for nurses, midwives and nursing associates*. 2015. <https://www.nmc.org.uk/standards/guidance/raising-concerns-guidance-for-nurses-and-midwives/>

[2] Nursing & Midwifery Council. *The code: Professional standards of practice and behaviour for nurses, midwives and nursing associates*. 2018. <http://www.nmc.org.uk/globalassets/sitedocuments/nmc-publications/revised-new-nmc-code.pdf>

**Supplementary Table 2.** Records identified through different database

| **Search number** | **Medline (through PubMed) Query**  **(The search was conducted in May 2022 (Italia) in Title and Abstract.)** | **Results** |
| --- | --- | --- |
| 7 | (“missed nursing care”[Title/Abstract] OR “unfinished nursing care”[Title/Abstract] OR “rationing of nursing care”[Title/Abstract] OR “prioritization process”[Title/Abstract]) AND “nursing students”[Title/Abstract] | **5** |
| 6 | “Missed nursing care”[Title/Abstract] OR “unfinished nursing care”[Title/Abstract] OR “rationing of nursing care”[Title/Abstract] OR “prioritization process”[Title/Abstract] | 507 |
| 5 | “**prioritization process**”[Title/Abstract] | 169 |
| 4 | “**rationing of nursing care**” [Title/Abstract] | 72 |
| 3 | “**unfinished nursing care**” [Title/Abstract] | 36 |
| 2 | “**missed nursing care**” [Title/Abstract] | 271 |
| 1 | “**nursing student**”[Title/Abstract] | 2579 |
| **Search number** | **CINAHL (EBSCO) Query**  **(The search was conducted in May 2022 (Italia) in Title and Abstract.)** | **Results** |
| S7 | S1 AND S6 | **3** |
| S6 | S2 OR S3 OR S4 OR S5 | 202 |
| S5 | TI “**prioritization process”** OR AB “**prioritization process”** | 3 |
| S4 | TI “**rationing of nursing care”** OR AB “**rationing of nursing care”** | 32 |
| S3 | TI “**unfinished nursing care”** OR AB “**unfinished nursing care”** | 19 |
| S2 | TI “**missed nursing care”** OR AB “**missed nursing care”** | 151 |
| S1 | TI “**nursing student”** OR AB “**nursing student”** | 10.312 |
| **Search number** | **Scopus Query**  **(The search was conducted in May 2022 (Italia) in Title and Abstract.)** | **Results** |
| 7 | #1 AND #6  ( ( TITLE-ABS-KEY ( “missed nursing care” ) ) OR ( TITLE-ABS-KEY ( “unfinished nursing care” ) ) OR ( TITLE-ABS-KEY ( “rationing of nursing care” ) ) OR (TITLE-ABS-KEY ( “prioritization process” ) ) ) AND ( TITLE-ABS-KEY ( “nursing student” ) ) | **5** |
| 6 | #2 OR #3 OR #4 OR #5  ( TITLE-ABS-KEY ( “missed nursing care” ) ) OR ( TITLE-ABS-KEY ( “unfinished nursing care” ) ) OR ( TITLE-ABS-KEY ( “rationing of nursing care” ) ) OR( TITLE-ABS-KEY ( “prioritization process” ) ) | 1151 |
| 5 | TITLE-ABS-KEY(“**prioritization process”**) | 781 |
| 4 | TITLE-ABS-KEY(“rationing of nursing care”) | 83 |
| 3 | TITLE-ABS-KEY(“**unfinished nursing care”**) | 45 |
| 2 | TITLE-ABS-KEY(“**missed nursing care”**) | 295 |
| 1 | TITLE-ABS-KEY(“**nursing student”**) | 34.905 |

**Legend:** CINAHL: Cumulative Index to Nursing and Allied Health Literature.

**Supplementary Table 3.** Records identified through database screening

| **DATABASE** | **Number** |
| --- | --- |
| Medline (through PubMed) | 5 |
| CINAHL (EBSCO) | 3 |
| Scopus | 5 |
| Duplication Studies | -8 |
| **TOTAL** | **5** |
|  |  |
| ***First Step (Title-Abstract-Keywords)** | **5** |
| Excluded studies | -2 |
|  |  |
| ***Second Step (Full text)** | **3** |
| Other Sources | +4 |
|  |  |
| **Eligible Studies, Total** | **7** |

**Legend:** CINAHL: Cumulative Index to Nursing and Allied Health Literature

**Supplementary Table 4.** Studies investigating Unfinished Nursing Care among students

| **Author(s), Year of publication, Country, Affiliation** | **Aims, Study design,**  **Setting, Year of data collection** | **Sample and participants** | **Data collection process and methdos/instrument** | **Main findings** |
| --- | --- | --- | --- | --- |
| **Qualitative studies** | | | | |
| Dimitriadou et al.  2021  *Country:* Greece and Cyprus  *Affiliation:* University  Part of PhD Thesis (Supervisor  Prof. E.  Papastavrou) | To explore nursing students’ perceptions on issues related to missed care (missed care events, reasons, and outcomes) in Cyprus and Greece  *Study design:* inductive content analysis  *Setting:* all three- and four-year students of four universities (one public and three private) of Cyprus and from one university in Greece  *Year of data collection*: 2018 | All nursing students.  *Inclusion criteria:*  -three- and four-year students  -enrolled in an undergraduate nursing programme as regulated by the European directive 2013/55/EU  *Cyprus:* 229 students  *Greece:* 381 students | Open-ended questions:  -*What are the missed care events witnessed by nursing students?*  *-In their opinion, what are the reasons that contribute to the missed care?*  *-In their opinion, what is the impact of missed nursing care on patient outcome?* | *Missed care witnessed*   - *Safe care:* hand hygiene, principles of aseptic technique, prevention and management of pressure ulcers, use of protocol, prevention of falls, administering medications on time, adequate patent surveillance - *Daily activities and comfort:* body and oral hygiene, put clean sheets, feeding, sleep needs. - *Communication and support:* inform patient or family for treatments/procedures, education, prepare patient and family for discharge, psychological and spiritual support - *Respect and dignity:* privacy, holistic approach, timely response to request, informed consent. - *Needs assessment and documentation:* evaluation and monitoring of vital signs, monitoring of blood sugar   *Reasons that contribute to missed care*   - *Indifference/Lack of interest*: task considered less important, culture, routine habit, no motivation, dissatisfaction - *Lack of knowledge*: devaluation risks and impacts - *Workload:* time consuming, understaffing, burnout   *The impact of missed nursing care on patient outcome*   - *Nosocomial infection:* stomatitis, urinary tract infections, bloodstream infection (catheter-related), pneumonia - *Increase length of hospitalisation, readmission and mortality*: medication administration errors, pressure ulcers, patient falls   *Negative psychological and emotional status of patient*: dissatisfaction, patient not recommending hospital, agitation and denial of receiving treatment, frustrations, the patient afraid to speak |
| Gibbon & Crane  2018  *Country:* United Kingdom  *Affiliation:* University | To explore how exposure to missed care during a pre-registration adult field nursing programme influences student nurses' professional socialisation  *Study design:* qualitative approach (Braun and Clarke’s thematic analysis)  *Setting:* UK Higher Education Institute  *Year of data collection:* not reported | Final year undergraduate (adult field) nursing students  *First focus group:* 10 participants  *Second focus group:* 8 participants | Two focus group  Main questions (synthesis)  *The problems you just read in the scenario are very similar to the ones we here from other nurses and read about in the literature. What kinds of dilemmas do you have in terms of the provision of nursing care and the priorities you set in your everyday work? Can you give any examples?*  *What areas of care do you think are most affected (from setting priorities)? Can you tell us why?*  *How is care at bedside nursing allocated? How do you decide what needs to be done? (Probe: are there any criteria for such decision making?)*  *While there may be differences between wards and between specialities, to what extent would you see priority setting and the situation of not being able to complete all the care you planned as influencing your employment (career) intentions? (Probe: Are there some clinical environments in which care is left incomplete or delayed? If so, would it influence your decision to seek a job there?)* | Themes   - *Awareness.* Participants were aware of missed care, and the examples of care that frequently were missed resonated with those previously identified in the literature (discharge planning, patient teaching, surveillance, moving or ambulating patients, hygiene, maintaining intake, output documentation, changing dressings) - *Rationale.* The most frequently cited rationale for missed care was staff shortages, lone working, the competing pressures of too many priorities, pragmatic acceptance of MNC, ineffective teamwork, poor transmission of information, and problems with delegation - *Impact.* A number of participants commented on missed care as “not killing anyone,” again suggesting a pragmatic acceptance of missed care. The participants themselves reported that missed care negatively impacted their feelings about nursing and nursing care - *Strategies to avoid.* Participants reported teamwork, “round” for all patients, and routinised care as strategies to avoid MNC - *Influence of missed care on career aspirations*. The participants in this study were more likely to seek to choose to work in very busy environments, such as Acute Medical Assessment Units, or Accident and Emergency Departments, as they saw this as providing them with an enriching experience in a wide range of different patient groups that would enhance their knowledge and longer-term career aspirations   Exposure to missed care can be articulated as a pathway. As part of their professional socialisation, students observe that care is missed and seek to understand and rationalise this. This creates a dilemma for the student, who is tasked with resolving their cognitive dissonance to reduce their discomfort while simultaneously developing the skills of prioritising care to reduce negative consequences for patients. This results in pragmatic acceptance that care is missed, potentially leading to the next generation of nurses being exposed to missed care, with the potential that acceptance of missed care will become the socialised norm |
| Habermann et al.  2022  *Country:* Germany  *Affiliation:* University | To explore undergraduate nursing students' experiences of MNC in clinical placements in Germany  *Study design:* Qualitative study (Mayring’s content analysis)  *Setting:* students from three universities in Germany (Bremen, Mainz, Münster)  *Year of data collection:* 2017-2018 | Purposeful sample.  *Inclusion criteria*: students  - having completed at least an initial period of clinical experience  - willing to participate  - could be reached for personal instructions and data collection in the classroom  *Response rate:* 69/69 (100%)  *Gender:* not reported  *Age:* not reported  *Academic year*: 32 (46.4%) first year; 32 (46.4%) second year; 5 (7.2%) third year | Written online reports:  - definition of the phenomenon  - example that students had experienced themselves during a clinical placement  - to elaborate on its meaning for them  - to explain how they had dealt with it | Themes:   - *Experience of MNC (care not finished, care only partially given, care not done/omitted, care delayed).* Students reported as MNC: leaving completely out or only partly rendering care for patients’ or residents’ personal hygiene and comfort, teeth brushing and care of dental plates, showers, nail or skin care, not changing patients’ or residents’ pads and pants, a lack of information for patients and relatives, a lack of care, involving psychological assistance and comfort in times of patients’ or residents’ instability and grief, administration of medicines, necessary wound dressings and all kinds of prophylactic measures - *Meaning of MNC (negative feelings, learning opportunities, disregard of student status, violation of patients’ and residents’ dignity, disregard of professional standards).* Being involved in missed nursing care led to intrapersonal conflicts, expressed as self-doubts and feelings of guilt, fear, and helplessness. Several respondents referred to their neglected student status on the ward, leading them to conduct tasks that they were not yet confident in or forcing them to comply with excessive demands they felt they did not have the power and standing to successfully object to. Students interpreted occurrences of MNC also as negligence of patients’ or residents’ dignity and rights due to nurses disregarding professional standards - *Dealing with MNC (improving work, reporting mentors, asking for help, accepting the situation, speaking with friends/family, applying self-management strategies, speaking with patients/residents).* Having been part of rationing nursing care motivated some students to try harder to render good care and to support patients or residents. In several other cases, however, such reports did not lead to the provision of support. Moreover, registered nurses on the ward often did not provide help when approached for support by students. Instead, some nurses delegated too many tasks, interrupted students occupied with tasks they had already resumed, and even assigned tasks that students were not yet allowed to execute. Some students developed self-management strategies to deal with stressful events induced by events that did not render appropriate care. Several students offered apologies to patients or residents when they had delayed or omitted care   *General reflections of students (lack of resources, state of nursing, work organisation, insufficient management).* The powerless status as a student silenced many protests, as did the awareness of a predicament in nursing care. Some students realised that there are restricted possibilities for professional nursing standards due to a lack of staff and time resources. A respondent compared nurses’ work to ‘assembly-line work’. In two reports, students pointed to the potential of minimising occurrences of MNC by optimising workflow and self-management. One student pointed to the observation of frequent staff turnover caused by poor management, leading to situations with heightened potential for MNC |
| Kalánková et al.  2021  *Country:* Slovakia  *Affiliation:* University | To investigate how nursing students interpret the concept of rationed care  To explore students’ experiences of rationed care during practical training in Slovakia  *Study design:* qualitative research design (Braun and Clarke’s approach for thematic analysis)  *Setting:* students from three universities across Slovakia  *Year of data collection:* 2019 | Purposeful sample.  *Inclusion criteria:* students  - full-time  - of final-year undergraduate  *Response rate:* 18/18 (100%)  *Gender:* female 18 (100%)  *Age:* 23.5 (range 21-23)  *Academic year*: all the final year of the bachelor’s degree in nursing programme | Individual, face-to-face, semi structured interviews:  -quality of nursing care from the perspective of nursing students  -understanding the concept of rationed care, students’ experiences with elements of care regularly rationed in clinical practice, and students’ perception of possible reasons for rationing nursing care  -students’ perceptions of how the phenomenon influences patient safety, nurses’ outcomes and nursing students themselves | Themes:   - *Incomplete care is normalised*. Sub-themes:   - *Individualised care versus ritualised performance.* Highlighted discrepancies that students identified represented differences between ritualised care methods and individualised methods or organisational systems for care provision. In the context of rationed care, they reported a lack of time to meet the psychosocial needs of patients. Students evaluated communication, emotional support, and patient and family teaching, together with patient mobility and hygiene care, as the activities most frequently rationed   - *Medical instructions as a priority*. Rationed care was associated with more independent nursing activities that are not precisely controlled. The implementation of particular independent activities results from personal decision-making and the professional responsibility of the individual   - *Simply, it is not done, and no one deals with it*. Students reported “silent” acceptance of the situation   - *From forgetting to neglect*. If the nurses do not highlight and acknowledge the issue of the phenomenon of rationed care, there is a real risk that it becomes normalised - *Provision of impersonal patient care.* Sub-themes:   - *The nurse behind the bar—no time to build the relationship.* Communication with a patient is limited to the purpose of the intervention, and it does not relate to the patient’s experience or reaction. As reasons for rationed care, the students mentioned high nurse workload, lack of time resulting from staff shortages, more motivation in making a living rather than an interest in caring, and lack of privacy - *Existence of a hidden curriculum for practice placements.* Subthemes:   - *I do what they ask me to do.* When students evaluated their decision-making processes regarding rationed care, they mostly described that their decisions were based on medical instructions and the health status of the patient. This mirrors the decision-making process of nurses   - *Habit forming—from initiation to resignation.* Students gave some examples of how nurses discouraged them from taking their initiative   *I do not make decisions; what if I make a mistake?* One of the factors influencing the decision-making process of students is fear of the consequences and stress related to having insufficient knowledge and skills |
| Kalfoss  2017  *Country:* Norway  *Affiliation:* University | To explore graduate nursing and pastoral care student’s perceptions of MNC in Norway  *Study design:* exploratory qualitative design (Van Manen’s thematic analyses)  *Setting:* students from a university college in southeast Norway  *Year of data collection:* 2016 | Purposeful sample.  *Inclusion criteria:* students  - in post-bachelor’s degree  - in cancer, nephrology, pastoral counselling, public health, and in community health nursing  *Response rate:* 32/32 (100%)  *Gender:* female 30 (93.7%)  *Age:* range 30 - 60 | Six focus groups  Main questions:  - “*When you think of the concept of missing care, non-caring, or the opposite of what you envision as good care, what do you think about? Can you give some examples?”*  - “*What factors do you believe contribute to not being able to give good care? Can you give some examples?*” | Themes (and subthemes)   - *Labour Constraints* *(Workload,* which featured time restraints, being too busy and not being able to carry out one’s duties in a good way; *Inadequate staffing; Time,* connected to inadequate staffing and moral distress; *Difficult patients; Withdrawal* from difficult clients*; Technology,* in relation to calculating time allotted for carrying out specific caring activities) - *Organisational Constraints (Leadership qualities,* such as bad leadership as being related to the leader’s need to be liked by staff or poor leadership in relation to unethical behaviours which had the possibility to inflict harm; *Time allotment,* by administrative leaders to carry out activities; *Rigid system; Lack of caring philosophy and standards; Lack of coordination; Lack of opportunities for self-reflection)* - *Professional Constraints (Professional attitudes,* such as having ambitions which the client didn’t share, not creating opportunities for client decision making, or feeling uncomfortable when advice was not followed; *Self-awareness; Personality characteristics; Not genuinely present; Judgmental; Cultural insensitivity,* in relation to choices; *Relationship with colleagues,* for example, the need to ask colleagues for help) - *Communication Constraints* (*Not acknowledging* the client as a person; *Not listening* to the client; *Not asking questions* to the client; *Self-interest; Not advocating,* not voicing one’s own beliefs; *Labelling* clients, which was stigmatising) - *Emotional Strain* (*Powerlessness; Loss of professional identity; Fatigue,* as a form of apathy observed in their colleagues; *Self-protective behaviour; Irritability,* with colleagues and viewing colleagues as meeting their own needs) |
| Najafi et al.  2021  *Country*: Iran  *Affiliation*: University | To explore the lived experience of postgraduate nursing students regarding MNC  To understand this complex phenomenon and its various dimensions to find a way to develop strategies for managing and reducing it.  *Study design:* interpretive phenomenology study (Dicklemann et al.’s approach to analysis)  *Setting*: Master’s degree students  *Year of data collection*: 2019 | Purposive sample.  *Inclusion criteria*:  - attending the Master’s degree  - with acceptable clinical practice experiences  - having passed their internship program  - being employed as clinical nurses in teaching hospitals  *Response rate:* 10/10 (100%)  *Gender:* female 6 (60%)  *Age:* 30.6 (range 23-39)  *Experience, years*: from 1 to 12 years | Individual, in-depth, face to face semi-structured interviews:  -“*What comes to your mind when I say missed care? What is missed care like?*”  -“*How do you feel when missed care occurs?*”  -“*Can you give an example?”* | Themes:   1. *Unfulfilled care*. Experience of missed care as a missed opportunity that may even lead to death and catastrophic consequences for the patient. Sub-themes:    - *deterioration of opportunity* (delay in care, irreparable situation, necessity missed, missing circle in providing care, unsuccessful care). Participants stated that the patients needed some care, but due to working conditions, it was ignored    - *induction of death* (painful outcome, equivalent to death, pushing to death, irreparable outcome, negative wave). Participants perceived missed care as an early induction of a critically ill patient’s death, worsening of the patient’s condition, pushing him or her into a bad condition, prolonged treatment, and a catastrophe, which will cause many complications for the patient, nurses, and organisation    - *worthless conception of care* (ineffective care, inevitable event, normalisation, unnecessary care, impatience in providing care). At students’ discretion, they prioritised the care that had led to the missing of some important care 2. *Living in Limbo.* Ethical conflicts and challenges that may arise in relation to the patient, and the confusions and emotions experienced by nursing students during missed care. Sub-themes:    - *ethical conflict* (trampling on the patient’s right, discredit, discrimination in care, being questioned, immoral care, feeling worthless and inadequate, deception, and denial, ignoring error, lack of adequate information, and proper training). Some nurses do not report missed care to their co-workers for fear of being reprimanded during their shift    - *the turbulence of existence* (feeling of regret, mental struggle, emotional struggle, stressful feeling, impact on the nurse’s mind, concealment of truth and disappointment). These feelings accompany students even long after the shift is over |
| **Quantitative studies** | | | | |
| Palese et al.  2021  *Country*: Italy  *Affiliation*: University | To validate a tool measuring the UNC phenomenon and its underlying causes as perceived by students  *Study design:* validation study  *Setting*: students from three nursing programmes in Northern Italy  *Year of data collection*: 2018 | All nursing students.  *Inclusion criteria*: students  - attending their clinical rotation during the study period (≥ one week)  - in the last week of the clinical rotation in order to allow them to be familiarised with practice  - in hospital and community care settings  - willing to participate  *Response rate:* 737/1190 (61.9%)  *Gender:* female 596 (80.9%)  *Age:* 23.3 (CI 95% 23.0-23.7)  *Academic year*: 256 (34.7%) first year; 233 (31.6%) second year, 248 (33.6%) third year  Convenience sample of clinical nurses supervising students in their clinical rotations in a random sample of units of the nursing programmes  *Response rate:* 30/30 (100%)  *Gender:* female (22; 73.4%)  *Age:* 39.2 (CI 95% 35.9-42.6) | Unfinished Nursing Care Survey (UNCS) tool (Bassi et al., 2020) adapted to students by involving them  Administered online; three reminders | *Validation*: among students, part A retained 22 items (out of 38) and 18 items were retained in part B (out of 26)   - - Part A: Methods of the Mokken scale were applied according to the monodimensionality of the tool. The scalability coefficients for each item and for each couple of items were greater than the lower bound limit of 0.30, and in most cases, they were greater than 0.5, suggesting that the selected items constitute a strong scale   - Part B: According to the Confirmatory Factor Analyses (CFI 0.933; TLI 0.914; RMSEA 0.092 CI 90% 0.085-0.099, p 0.000), reasons for UNC were categorised into six factors: ‘Communication’, ‘Priority setting’, ‘Nurse’s aides’ supervision’, ‘Material resources’, ‘Human resources’, and ‘Workload unpredictability’. The internal consistency of part B was alpha 0.950   - Acceptability: Missed item Part A from 0 to 5.2%, part B from 0 to 7.2%   *Finding*s: Students ranked ‘Going to patients without being called’ as being more often unfinished and ‘Performing bedside glucose monitoring’ as being less often unfinished. ‘Human resources’ was reported as having the greatest impact on perceived UNC: moreover, ‘Workflow unpredictability’ was perceived as greatly affecting the occurrences of UNC followed by ‘Communication’ issues  Students with more experience reported lower perceptions of UNC. Interventions at higher risk of being unfinished have been reported to have significantly lower averages among third-year students as compared to second- and first-year students. Having attended a higher secondary school before the nursing programme and being supervised by a clinical nurse were positively correlated with perceived UNC. In contrast, the perceived adequacy of the nurses, and nursing aides, the duration of the clinical rotation, and higher achievement of the learning outcomes were reported to be negatively correlated with UNC  No statistical differences emerged between the students’ perceptions of UNC and that of their clinical supervisors |

**LEGEND:** CFI: Comparative Fit Index; CI: Confidence Interval; MNC: Missed Nursing Care; RMSEA: Root Mean Square Error of Approximation; TLI: Tucker–Lewis Index; UNC: Unfinished Nursing Care; UK: United Kingdom.
